# Exploring How and Why to Develop Patient-Centered Packaging: A Multiple-Case Study with Pharmaceutical Companies

**DOI:** 10.1007/s43441-021-00338-0

**Published:** 2021-09-28

**Authors:** Giana Carli Lorenzini, Annika Olsson

**Affiliations:** Packaging Logistics, Department of Design Sciences, Lund University, Box 118, SE 221 00 Lund, Sweden

**Keywords:** Multiple-case study, Packaging development, Patient-centered packaging, Pharmaceutical industry, Workshops

## Abstract

**Background:**

Patient centricity has gained attention ranging from regulatory authorities to patient advocacy groups, calling for pharmaceutical companies to revise their traditional business approach to drug development by including the development of solutions that are meaningful in patients’ lives. Medication packaging is one area where empirical evidence is lacking about the incorporation of patient centricity. This study aimed to explore patient centricity applied to pharmaceutical companies’ packaging, and to identify the specific challenges faced and lessons learned when developing patient-centered packaging.

**Methods:**

The study followed a multiple-case study research approach based on five cases of patient-centered packaging development in mid- and large-sized pharmaceutical companies.

**Results:**

Patient-centered packaging is often associated with the intuitive and self-explanatory use of the medication by patients. Patient-centered packaging comes with challenges, but also offers opportunities for the creation of better solutions for patients and learning for the teams involved. To overcome these challenges, it is essential to build a business case that justifies such development, one where patient needs are present from the start and aligned with other imperative deadlines of drug development, with stakeholders onboard.

**Conclusion:**

Patient-centered packaging is the exception rather than the norm in packaging development due to a conventional approach where packaging plays an ancillary role to drug protection. The cases presented here challenge this approach and can inspire other companies to carry out patient-centered packaging development. The cases are also relevant to other actors who are interested in continuously promoting the dialogue about patient centricity in healthcare.

## Introduction

The pharmaceutical industry is a research and development (R&D) intensive industry. It is also highly regulated for the safety and efficacy of the new drugs it launches in the market, and reliant on patents and market exclusivity for innovation [1]. This business model has been almost intact for decades [2]. Developments in recent years, however, have challenged the way the pharmaceutical industry has established itself: loss of patents, limited promotional practices, rising costs for R&D, increased rigor in the regulatory environment, flatlined pharmaceutical outputs with fewer new medications reaching the market, increased scrutiny from healthcare payers on costs and the value of medication [3, 4]. Among all these aspects, the traditional view of patients having a secondary role in decisions taken on their behalf regarding their treatment has been contested [5]. Patients have become empowered through disease-specific patient advocacy groups, social media, and online platforms [6–8]. In connection with that, medications that previously were paid for when sold are now considered to be paid by performance, indicating that pharmaceutical companies may only be reimbursed when patient outcomes improve [9].

The current challenges shed light to patient centricity, calling the pharmaceutical industry to act by designing meaningful experiences for and with patients in their treatment, for which adherence is paramount [10]. Patient centricity needs to be systematically applied to the design of pharmaceutical drug products. This relates to many elements of the medication regimen, for instance, how the drug is to be taken (e.g., orally or intravenously), when and with what sort of auxiliary tools (e.g., dosing devices, instructions for use) [11].

Medication packaging is one key element that has been overlooked in terms of patient-centered pharmaceutical drug product design. The expected shift to pay-for-performance and the attention to patient outcomes means packaging has the potential to contribute to improved patient experiences and outcomes in medication adherence and treatment. Medication packaging is the vehicle that protects the drug on its journey from manufacturing to the hands of patients around the globe [12]. Medication packaging and its components (i.e., labelling, enclosed leaflet, and additional texts) also provide information that stays with patients in self-care, for instance, when the physician and other healthcare providers are not available to tell them about the drug intake and storage. Finally, medication packaging can have important utility functions that provide cues to patients to support them in adherence to treatment, such as added calendar features that let patients see when a tablet has been taken [13].

Despite its multiple functions, a study with stakeholders from the pharmaceutical industry shows that the traditional view of packaging to protect the drug prevails, to the detriment of other important functions for patients [14]. Overall, patient needs are repeatedly put aside among the many trade-offs in the decision-making process of developing medication packaging. As a result, medication packaging often becomes a burden when it adds too many unnecessary steps in drug intake or when it imposes several functional difficulties to patients [15, 16]. Additionally, medication packaging has been reported as a source of confusion, leading to medication errors with severe clinical consequences [17]. Nevertheless, to date there is a lack of empirical evidence about the development of patient-centered packaging by pharmaceutical companies and the challenges of doing so.

## Study Objectives

The objectives of this study were: to explore patient centricity applied to packaging development by pharmaceutical companies, and to identify the specific challenges faced and lessons learned when developing patient-centered packaging. We purposefully selected and analyzed a series of cases in the pharmaceutical industry where patient centricity had been applied to packaging development, or where conscious efforts had been made to attend to patient needs in relation to the packaging and delivery of the drug.

## Background

### Patient Centricity

Patient centricity has been called for from different stakeholders and perspectives, from regulators to patient advocacy groups. Since 2014, the U.S. Food and Drug Administration (FDA) has made efforts to establish the grounds for patient centricity, by discussing with them the impact of diseases in their lives and “how to develop more effective and user-friendly medical interventions” [18]. The Patient Engagement Advisory Committee (PEAC) is one of the FDA’s efforts. It is composed of patients, caregivers, and representatives of patient advocacy groups in discussing relevant issues that affect patients [19]. The focus is to increase the influence and level of participation of patients in decisions that directly affect their healthcare, from clinical trials to delivery of treatment [20].

Research on patient centricity is fairly recent, with most of the scientific literature coupled to studies reporting on patient centricity in clinical trial processes (e.g., Gregg et al. [21]), whereas studies are scarce that present the pharmaceutical industry in this area. According to the literature, patient centricity means to put patients first by partnering with them from discovery, research, development, distribution to access to medicines, all of this aiming to achieve better patient outcomes [22]. As emphasized by scholars, patient centricity implies an organizational shift for pharmaceutical companies from a disease-centered to a patient-centered strategy, where “patient well-being is placed at the core of all initiatives” [23]. Similarly, Robbins et al. [24] explain that patient centricity is larger than empowerment or engagement: it implies that “the patient is at the center from the start and remains there”, and that the patient is supported with the necessary tools to make informed decisions about their treatment.

Barei [25] sheds light on patient centricity as a path for pharmaceutical companies to add value to already existing medicines. This should be done in a combined effort to better meet patient needs and strengthen their position on the market. A value-added strategy focused on patient centricity should then consider improvements in safety, adherence, as well as appropriate pharmaceutical design aspects, which include design of the drug, its route of administration, as well as its packaging.

Katsanis et al. [26] explain that patient centricity is composed of three interconnected elements: patient adherence, patient outcomes, and patient experience. Patient adherence refers to the degree to which patients follow the treatment and continue to do it over time, as prescribed. Lack of patient adherence implies additional costs for the healthcare system, but also impacts patient outcomes when the expected response to a treatment is not achieved. Patient outcomes also relate to general well-being and quality of life. Finally, patient experience, also known as patient journey, refers to “all the sequential phases in providing a patient’s care and may include both clinical and non-clinical steps” [26]. The patient experience is affected by the many interactions among different stakeholders involved in healthcare, from the manufacturers of the drug to physicians and other supportive systems that surround the patient in their treatment [27].

### Patient Centricity Applied to Medication Packaging

Medication packaging has been traditionally product-centered with an emphasis on drug protection, but limited in consideration of a patient-centered perspective [28]. The consequences of such an approach have been studied by a number of scholars. Difficulties opening packaging are among the most common functional problems faced by patients. As reported in the literature, this is accentuated, for instance, for older females due to reduced hand strength, and patients with conditions that impair their dexterity [29–31].

Compared with other sorts of packaging, medication packaging is highly regulated and extensively tested to guarantee medication integrity and protection on its journey from the manufacturer to the hands of patients around the globe. There are limited opportunities for marketing exposure and branding [12], for instance, as described by the U.S. Food and Drug Administration [32]. Regulations in this area have also created many classical trade-offs that are well recognized by the industry, but that are still difficult to solve [33]. An example is the need to have child-resistant (CR) features and still be senior-friendly (SF), as established by the U.S. Poison Prevention Packaging Act of 1970 [34, 35]. In Europe, regulations requiring a unique identifier and an anti-tampering feature on the outer packaging of medicines aims to increase security levels and safety levels through the supply chain until the medication reaches the hands of patients (Directive 2011/62/EU) [36]. Yet, these added features can also impact the accessibility to medications by patients.

## Methods

This study followed a multiple-case study research approach. Case studies are context-dependent and relevant when the researcher aims to answer *how* and *why* questions that lead to an understanding of a phenomenon [37]. Specifically, we focused on two overarching research questions: How is patient-centered packaging developed? Why is it a challenge to do so?

A multiple-case study is based on theoretical sampling, intending to make cross-case comparisons using varied empirical evidence, making the results robust and replicable [38]. Cases are carefully selected because they can either predict similar results or anticipate contrasting results [37]. This differs from a single-case study, where the focus is on the uniqueness or representativeness of a concrete example to theorize about the phenomenon.

It is well-known in the pharmaceutical industry that packaging is often designed with a traditional view of drug protection. This can hinder the adaptation of packaging design to meet patient needs. In our study, five unique cases of patient-centered pharmaceutical packaging developed by pharmaceutical companies were purposefully selected. The cases encompassed the primary packaging system level [39], which protects and delivers the medication used by patients. This system is assessed by regulatory authorities and includes both the inner packaging in direct contact with the medication (e.g., blisters, glass vials) and outer packaging containing the inner packaging (e.g., carton board box).

### Sample of Companies

The selection process of the pharmaceutical companies targeted the ones that were well-respected and acknowledged for their drug development. As we were aiming for packaging projects, it was also important to focus on companies that have publicly addressed their interest in patient centricity. To achieve that, industry reports with top-ranked pharmaceutical companies were collected and read. In addition, one researcher was responsible for attending pharmaceutical and packaging oriented online events where pharmaceutical companies gathered and/or were awarded for their patient-centered packaging (e.g., Pharmapack). Finally, we also searched for key contact points through professional social media platforms (e.g., LinkedIn). Five of these companies fit the purpose of the study and accepted to participate (Table 1). Due to privacy requests from the companies, participants’ names were omitted, and the companies referred to as Case A, Case B, Case C, Case D, and Case E.

**Table 1:** Profiles of the companies

Case	Respondent’s position	Company profile	Number of employees
Case A	Head of Operations, Global Healthcare Operations, Connected Health and Devices	Large pharmaceutical company Global operations in more than 60 countries HQ in Europe	> 40,000
Case B	Director of Drug Product Development, Drug Design and Development Director and Head of Packaging and Investigational Medicinal Product Operations, Global Manufacturing Packaging and Investigational Medicinal Product Manager, Global Manufacturing	Medium biopharmaceutical company focused on rare diseases Global operations in more than 30 countries, delivering treatment in over 70 countries HQ in Europe	< 2000
Case C	Senior Packaging Engineer Senior Human Factors Engineer Packaging Engineer Senior Packaging Engineer	Large pharmaceutical company Global operations in more than 100 countries HQ in Europe	> 40,000
Case D	Senior Manager Materials, Microbiology, Packaging and Sustainability Packaging Designer	Large pharmaceutical company Products sold in more than 180 countries HQ in Europe	> 40,000
Case E	Secondary Packaging and Artwork Development Leader Packaging Industrialization Leader	Medium biopharmaceutical company focused on severe diseases Global R&D, Marketing and Sales platform: Operations in more than 40 countries HQ in Europe	> 7000

The five companies were selected for the following reasons: (a) they were mid-/large-sized ethical pharmaceutical companies and top performers in their industry; (b) all companies had established processes and allocated teams for packaging development; (c) each company had developed at least one patient-centered packaging for which they could disclose to the researchers the history of the development process, and provide additional data from different sources; (d) participants had several years of experience in pharmaceutical packaging projects, many of them with strategic and managerial positions in the company. They were also responsible for the development of the patient-centered package and could join in an online workshop with team members and report their experiences from that development.

### Data Collection and Analysis

Once a key contact person was identified, the first contact was made via email, phone, or messaging on a professional networking platform. One digital document was sent with the description of the study and the topic areas that would be addressed.

Data were collected mainly through online workshops via Microsoft Teams, held separately with each company. The companies decided who would be the relevant people (*n* = 1–4) to participate and which case of patient-centered packaging to address. For each workshop, a *Miro* online collaborative whiteboard platform was customized [40], with semi-structured questions on main thematic areas to discuss. The participants were given access to the online board one week ahead of the workshop date. On the day of the workshop, the host researcher started the meeting, welcoming the participants and briefly introducing the purpose of the study. Participants had the opportunity to ask questions. During the workshop, participants and the host could simultaneously add content to the online whiteboard. Each workshop took on average two hours, was recorded, and transcribed.

After the workshop, the researcher (who was also the host of the workshops) sent a high-quality image of the *Miro* whiteboard to the participants for a final check. Amendments and additional information could then be added. Further information was also gathered from other sources, such as pharmaceutical industry reports, companies’ websites, internal documents provided by the participants regarding the packaging concepts developed and their approach to patient centricity. Due to confidentiality issues, internal documents, images of packaging concepts, as well as the final *Miro* boards cannot be displayed publicly here.

A data analysis was performed using a traditional open coding process [41] to examine and categorize all the data. Additionally, a copy of each interactive whiteboards was created and imported to a common board, where all the responses were compiled and compared according to their content. Duplicates were removed and similar information grouped. Table 2 presents a summary of the five cases.

**Table 2:** Overview of the five case studies

Cases	Background on case	Characteristics of the drug	Route of administration	Level of innovation of the drug
Case A	The medication was launched in the market, but there were difficulties identified with the openability of the current packaging. A new packaging solution was investigated to facilitate access to the medication with easy opening, also making it possible for patients to take the tablets without touching the drug	High toxicity of the medication requires care when patients handle it	Non-parenteral, oral tablets To be taken by the patient	Innovative drug, permitting patients to have shorter periods of treatment No self-injection when compared to other products in the market
Case B	First product developed by the company in a market that was already competitive for the illness, and first time the packaging was developed from the start. The project was an opportunity to develop a packaging system “from scratch”, but also build trust and recognition when reconstructing the drug for application focused on the innovation of treatment with reduced injections and convenience to patients	Stored in the refrigerator	Parenteral Medication needs to be reconstituted by the patient for self-injection To be taken by the patient	Reduced number of injections necessary when compared to other products in the market
Case C	An opportunity arose to continue the packaging development initiated for another product to this product instead. Openability features were the focus of the development to facilitate easy removal of a pre-filled syringe from its outer box. There was a possibility to create a platform for other similar packages	Stored in the refrigerator	Parenteral Pre-filled syringe for self-injection To be taken by the patient	Standard syringe First and only approved subcutaneous medicine for both adults and adolescents with the disease
Case D	The company wanted to launch an oral tablet in the U.S. market that would require packing it in a CR/SF package. The characteristics of the drug demanded exploring alternative blister packs to protect it from moisture, and a strong outer box for child-resistance. Oral tablets were not the main expertise in the company at the time of development. There was a possibility to create a platform for other similar packages requiring CR features for blisters	Moisture sensitive drug, which needs to stay in its original packaging	Non-parenteral Oral tablets To be taken by the patient	Highly innovative drug to be taken once a day. It can be a substitute for other drugs that would demand injecting the medication
Case E	The drug product packaging was being renewed, which offered an opportunity for renewing and improving the usability and readability of information in the packaging and to improve patient adherence. This is the leading product for the company in the U.S. market, which demanded cautiousness in the changes to be made as healthcare providers and patients already knew the product. There was a possibility to create a platform for other similar packages	Stored in the refrigerator	Parenteral Pre-filled syringe for self-injection To be taken by the patient	Incremental change. It was first launched to be administered by a healthcare professional

## Results and Discussion

The analysis of the five cases shows the choices made along the way by each company not only to motivate the development of a patient-centered packaging, but also to engage relevant stakeholders in the conversation for patient centricity and to overcome challenges along the way. Table 3 presents each packaging concept developed by the companies, the user studies carried out, the allocation of responsibilities, and the packaging concept’s status in the market.

**Table 3:** Development of packaging concepts from the five cases

Case	Packaging concept	User studies	Responsible for designing the packaging	Teams involved in the packaging development project	Responsible for the final decision	Status in the market
Case A	Pot containing tablets with a sliding lid. Several pots were assembled in an outer box as an alternative concept to a blister pack CR features added to the pot and outer packaging	Patients with dexterity impairments (*n* < 15) received two packaging concepts at home and videotaped themselves interacting with the packages	External packaging design consultancy (from ideation to design for manufacturing)	Core team: – Manufacturability, Technology (design requirements) – Project Manager – Regulatory Affairs – Quality Assurance – Human Factors (user study) Extended team: – Supply of the current product (to compare the new concept with the concept already in the market)	Marketing and Commercial	Project reached proof of feasibility, and then was stopped Packaging in the market is a blister packaging with CR features
Case B	Outer box containing several components in the packaging system (e.g., glass vial, sterile powder, one syringe)	Used two different routes: (1) outspoken needs in focus groups with patients with the disease and healthcare professionals (*n* > 100); (2) video diaries based on observations of patient behaviors at home and several iterations based on one early prototype	External packaging design consultancy + packaging supplier with guidance of the project team	Project team composed of different areas: – R&D – Commercial – Production – Regulatory Affairs	Project team made the final decision Joint decision	Approved. In the market
Case C	Outer box with card insert inside containing one pre-filled syringe	Pre-study: the core team had a test scenario to prove that the right forces were feasible for the packaging (balancing user and technical requirements) Human factors testing with patients (*n* < 10) in the very beginning for error detection, followed by prototype testing with small samples of representative patients	Packaging supplier with guidance of the Global Device Development team	Core team: Global Device Development team composed of multidisciplinary roles, including Human Factors Supportive teams (e.g., Verification Engineering)	Global Device Development team makes a recommendation for the packaging concept Stakeholders move the recommendation forward	Approved. In the market
Case D	Aluminum foil 30-day blister pack attached to an outer box with CR features Three of those boxes are placed inside a larger box	Pre-study to see patients interacting with a dozen existing CR packaging concepts Patients rated the packs CR/SF standard test according to U.S. protocol involving children and older people; 4–5 tests performed until they got a pass with the package concept	Blister packaging: developed internally by the primary packaging department Outer box for the blister: patented concept from a packaging supplier, altered with guidance of the packaging development team	Core teams: – Packaging Development (for secondary packs) – Labeling and Graphics – Production/Manufacturing Development Supportive teams: – Marketing – Usability/User Communication – Strategic Sourcing	Collective decision: the stakeholders rated 2–3 final concepts until reaching consensus for a final one	Approved. In the market
Case E	Starter kit for new patients composed of an outer box containing card insert and three inner boxes with two pre-filled syringes each	User study with a blank packaging to test features first. Incorporated user data from a previous study Online survey with patients (*n* < 70) about their perceptions of the new packaging	External packaging design consultancy + packaging supplier with guidance of the Secondary Packaging team	Core Team: – Secondary Packaging – Industrialization Supportive teams	Collective decision within the core team Marketing and Commercial approved final visual graphic identity	Approved. In the market

Despite the idiosyncrasies of each case, common grounds were identified and are thematically discussed here according to the following four themes: designing packaging that enhances patient outcomes, involving patients and translating patient needs into packaging functionalities, building the case for patient-centered packaging, overcoming challenges for patient-centered packaging and learning from it.

### Designing Packaging that Enhances Patient Outcomes

All cases have medication intended to be administered independently by the patients at home. Yet, these medications have a higher level of complexity and refer to treatment that needs to be followed for weeks or even for a lifetime. Therefore, in the five cases, patient centricity is aligned with the self-explanatory and intuitive use of the drug communicated by the packaging.

**Fig. 1:**
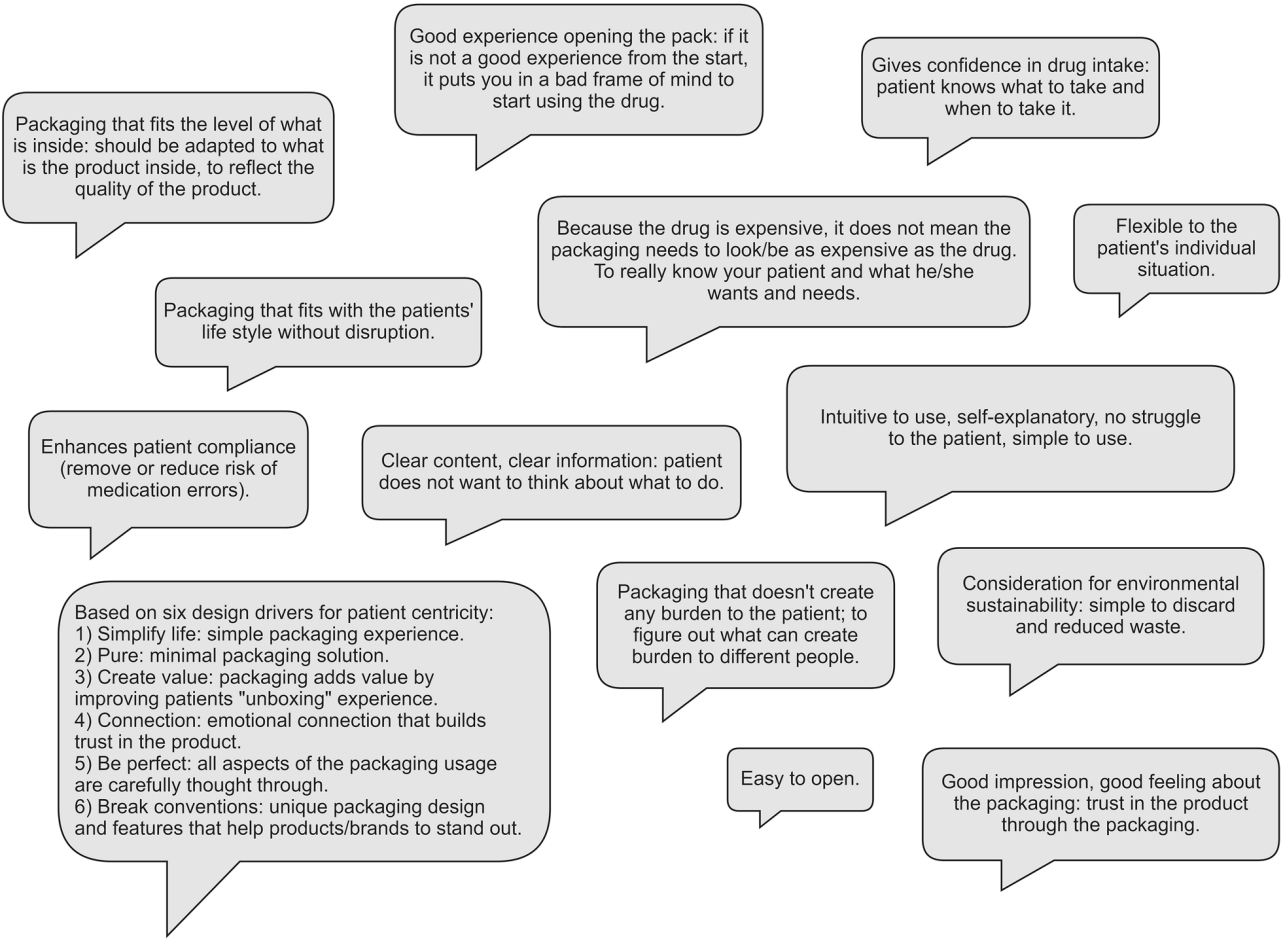
Collection of definitions of patient-centered packaging in the workshops

The view of patient centricity that is generally applied is related to the extent of convenience in the use of the drug, with packaging as the means for improved patient outcome. An example of this is permitting patients to have shorter treatments, fewer injections, or all the components for taking the drug in a package that can be easily carried *on-the-go* (Fig. 1).

These views are similar to the general views of patient centricity found in the literature [11]. Patient-centered packaging smooths the process of taking the medication and helps patients to build confidence that they are doing it right. Patient-centered packaging should also fit the lifestyle of patients without creating disruption in their routines or habits, as explained by one participant:

*It fits with their lives, like it fits with their fridges. The box or the bottle has an appropriate size that can fit in a medicine cabinet, or blister packs that fit into their purse. Whatever it is, we [need to] understand the different touchpoints where there is an opportunity to fit into the patient’s life without being disruptive. (Participant, Case C)*

To do that, easy opening is also considered relevant as in all the cases openability features were investigated:

*From a patient perspective, really struggling to open, almost destroying the pack to get into it automatically puts you in a bad frame of mind to start injecting yourself or whatever it is that you have to do. (Participant, Case D)*

It is significant that patient-centered packaging needs to show robustness to create trust in patients about the quality of the drug product. However, a balance is needed to avoid giving the impression that the company is lavishly using its monetary resources for developing the package instead of the drug. Excessive use of materials that can add unnecessary steps, or packages that are difficult to discard post-use should also be avoided.

*If you buy something high-end, it’s fine that the packaging also feels robust and nice. There is a nice feel, and you can see it’s not cheap packaging. But if you buy a product that is small or cheap or something you use a lot, then you also need to adjust the packaging so that it’s smaller and maybe less robust, so you also don’t feel you are wasting too much packaging. So, it’s always a balance with the product inside. (Participant, Case D)*

The respondents were asked about the importance of packaging in helping patients to carry out their treatment and about the technological changes that have occurred. The response was that packaging was a source to online channels or augmented reality tools, creating an ecosystem for patient guidance and information. In the five cases, this was done via the use of QR codes to access informative company’s websites, links to educational videos, and telephone numbers to reach out for healthcare support in the use of the drug. Enhanced communication by means of interactive packaging is a trend not be ignored [42]. Critical thoughts are necessary, however, as some patients may be excluded from accessing relevant information if they have limited access to, or limited literacy in the technologies used (such as the QR codes). Thus, additional research focusing on how companies balance these limitations and educate patients to use the technology applied to packaging would contribute better knowledge on patient-centered technological features.

### Involving Patients and Translating Patient Needs into Packaging Functionalities

Developing patient-centered packaging means to incorporate patient needs from early stages into functionalities in the packaging. This translates by involving patients in the process to better understand how it is to live with the illnesses [10]. Patient needs were considered along the entire packaging development process. This differs from other projects where demands from other stakeholders are upfront and are technically driven.

*In some cases, we don’t consider the user [patient] to a larger extent. It’s primarily product-focused, and we do not really consider the user [patient] unless it is justified. (Participant, Case B)*

For most of the cases, this also meant to study patient behavior even before starting to design the packaging. Respondents highlighted the importance of having patients involved to express their own needs, but also to observe these patients interacting with early prototypes to visualize how they would open the packages, acquire information, and interact with the drug:

*We tried to use two different routes: one was to talk with some patients about what is the best packaging; another way was to passively observe and discuss with them [the patients], ask them to tell us how they use their treatment and, through that, understand potentially what the needs are. (Participant, Case B)*

Involvement of patients requires additional time and resources and is often performed qualitatively through several iterations by collaborating with patient advocacy groups, but also with design partners and packaging suppliers. In the workshop sessions, the respondents commented that these iterations resulted in some features not being further pursued, or to be consistently altered. It also became evident from the cases that the final decisions about a packaging concept may be taken internally among multidisciplinary packaging teams because many of the requests from patients do not comply with regulations, nor do they consider a holistic view of how the packaging will be produced.

### Building the Case for Patient-Centered Packaging

Packaging has often an ancillary role in the drug development process and is usually developed when drug formulation and dosing forms are already defined. For dosing forms established within the company, preference is given to packaging already developed and approved as this is also cost-efficient [14]. This implies that there should be a reason for spending more time and resources in patient-centered packaging development. Respondents from the companies addressed it as *making a business case* out of a very specific packaging development, and engaging relevant stakeholders in the packaging development process.

This mirrors what has been previously found in the literature, firstly about designing products to better meet consumer needs [43], and secondly to integrate product and packaging development processes from the initial phases and onwards [44].

*The reason why we decided to go for this innovation was because we did some [user] studies, and someone said: “I feel stupid, I cannot open my medication”. That is not really what you want, right?! So, to answer your question, indeed, this packaging was different than anything we have had. (Participant, Case A)*

As explicitly commented on in Case E, it was necessary to advocate internally and defy traditional views of packaging development. It differed in Case D, where it was important to communicate well with the external packaging supplier to align their views about the packaging concept. As for Case A, their packaging concept could not continue because “packaging was not discussed early enough” to build their case internally and to create a good fit with the current marketing strategy for the product.

In all cases, the patient-centered packaging was not only an opportunity to improve packaging for patients, but also to increase their market share and visibility. Moreover, there was an intention to use those cases internally as a platform for other similar packaging development. For instance, in Case D, they established some boundaries for blister development with CR/SF requirements that were new to them. In Case C, they created the basis for similar packaging with a top opening feature that had a syringe placed inside.

### Overcoming Challenges for Patient-Centered Packaging and Learning from It

Developing packaging, in general, is not without challenges. Some of these challenges are ordinary to any packaging development process, for instance, complying with the existing timelines, or having a package that runs smoothly in the existing machinery.

*We have molecule-driven timelines, and it is not acceptable to slow down therapies that are gonna be life changing for a lot of people because your pack is going to be a little bit hard to open. I am a Human Packaging Engineer, and I want it to be as easy to use as possible for the patient, but you can imagine these people that spent decades developing this drug, and they want to see it go to market as quickly as possible, because they really do care about the patient, […] sometimes you have to sacrifice certain things. […] So, it is up to us to do this development outside of the molecule, which is also hard because maybe you are not able to get the funding, interest, or the resources. (Participant, Case C)*

*If you want to be able to do something like this, you need to start in parallel to everything else when you develop the drug, because it does take time. It is easy to neglect these things. And when you run out of time, you just put a vial in a box. I think it is valuable to respect that it does take a very long time if you want to do this properly. (Participant, Case B)*

For patient-centered packaging, however, the question that then arises is how to balance these challenges and not end up prioritizing all other requirements to the detriment of patient needs [14].

*At the end of the day, we need to be able to produce those packs. And if it’s about millions of syringes, you cannot pack them all by hand. So, you need to find a balance between [patients’] impairments and production constraints. Because what is best for the user [patient]? Is it to wait for the perfect pack or to be able to be served with a less perfect pack, so that at least you have access to the product? (Participant, Case E)*

Considerations about additional cost of a package can also shadow patient centricity. The cases here differed from the ordinary as the drugs to be packed were rather expensive prescription drugs, providing innovative treatment regimens for which there were good market opportunities.

As reported in the literature, there are still regulatory requirements on medication packaging that perpetuate the classical trade-offs in packaging development [35]. One example is to create robust packaging that can travel across the globe, but that is still easy to open when it reaches the hands of patients. However, it also became evident from the data that there is a call for patient centricity in packaging from regulatory authorities [19].

*The key learning was that the FDA has really changed the way they look at packaging in the past decade. This pack would have been totally impossible to imagine 10 years ago, and now it went through the whole process without one remark. This means a lot of hope for patient-centered packaging. But also means that you need to build the supportive data showing it’s driven by user feedback. (Participant, Case E)*

Patient-centered packaging may also mean establishing collaboration with external packaging suppliers and/or design consultancies. This is because pharmaceutical companies tend to focus on the R&D of drug development while external partners can dedicate efforts to designing new packaging solutions that overcome some of the forementioned trade-offs [33]. Partnerships were identified in all the cases. Case D, however, was different, as partnering with an external packaging supplier was rather the exception than the norm for their packaging development:

*The main difference was that we were using somebody else’s packaging from the start. We usually develop our packages from the ground up to suit our needs. […] And because it was their packaging concept, it added an extra element of frustration and difficulty. We couldn’t just change things. They still owned the patent. They were sometimes very reluctant to make the changes we asked for because they thought it was not a good idea. You know, in their minds, they thought we were making a mistake. (Participant, Case D)*

In general, from the participants’ answers, it seems that packaging development is never challenge free. Despite that, patient-centered packaging was also perceived as an opportunity to come up with “*a good packaging solution*”:

*I wouldn’t say it was more challenging. It was actually offering more changes for coming up with a good packaging solution. For sure, it also comes with work, but very meaningful work. Maybe the challenge was to find new ways. But to be honest, I also think this is daily business. We need to be flexible and find new solutions for each project. I wouldn’t say that we have this “typical project”. I would rather say each project is a little bit different and comes with its own challenges. (Participant, Case C)*

Furthermore, it became evident that even though the cases provided opportunities for learning among the professionals involved, these cases are still inspiring exceptions in the overall development of medication packaging and may not be enough for a true change to patient centricity, as one participant expressed well:

*We demonstrated that we can, very quickly, come up with high-level feasibilities. I don’t know if we really shifted the dial significantly in how packaging is developed. I think it takes more than one experience like this. It’s a big company, it’s just so many people working on packaging in so many different areas… Most people don’t even know that we did this. (Participant, Case A).*

**Fig. 2:**
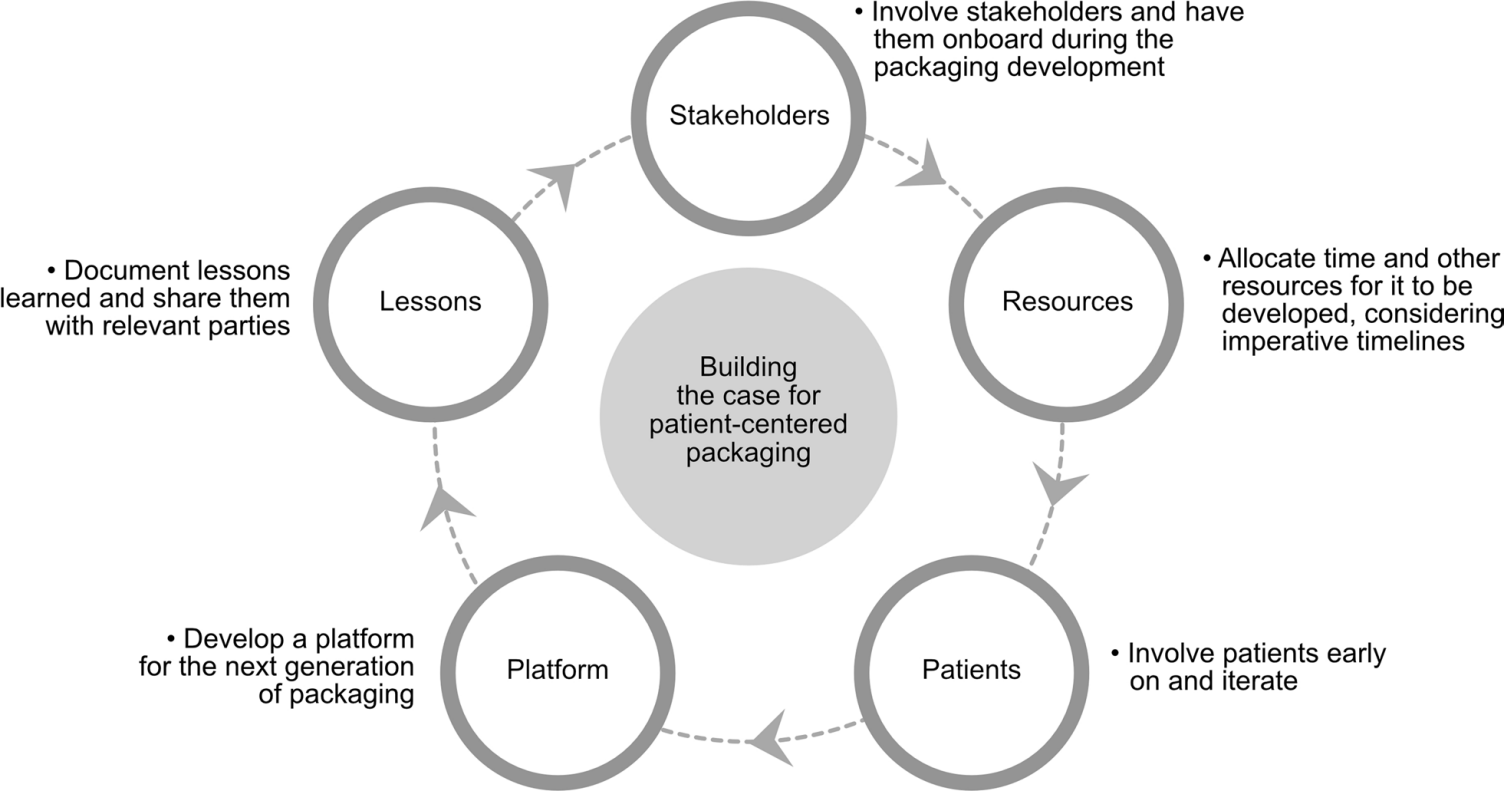
Building a business case for patient-centered packaging

A summary is graphically presented in Fig. 2 based on the main themes identified and discussed.

## Conclusions

Developing patient-centered packaging may not be the mainstream of packaging development for pharmaceutical companies because it is not embedded in their business model, where innovation is based on drugs that are later prescribed by physicians. This puts patients in a secondary role in any choices made on their behalf. Regardless of that, the cases presented here illustrate opportunities to alter the traditional approach to packaging as merely being a commodity, but instead viewing it as a tool to facilitate the intake of treatments by patients. Our results show the need to create a business case for patient-centered packaging. Ideally, this means bringing the patient perspectives into the process of designing the packaging early on, but also allowing time, resources, and engaging key stakeholders in this process. The companies also reported lessons learned from patient-centered packaging development. One recommendation is to document these lessons so they can be useful in other projects, and to share them with others in the organization.

Our results can inspire other companies to lead initiatives toward patient-centered packaging development, as well as continue to stimulate the debate where patient needs, and involvement are also considered in the update of regulation. Finally, further research can be carried out to continuously build a robust body of empirical evidence about industry practices towards patient centricity.
